# Cholestanol accelerates α-synuclein aggregation and spreading by activating asparagine endopeptidase

**DOI:** 10.1172/jci.insight.165841

**Published:** 2023-11-08

**Authors:** Ting Yu, Shuke Nie, Lihong Bu, Miao Liu, Juanfeng He, Xuan Niu, Hongyan Feng, Jifeng Guo, Beisha Tang, Zhaohui Zhang, Keqiang Ye, Haiqiang Jiang, Liam Chen, Zhentao Zhang

**Affiliations:** 1 Department of Neurology, and; 2 PET-CT/MRI Center, Faculty of Radiology and Nuclear Medicine, Renmin Hospital of Wuhan University, Wuhan, China.; 3 Department of Neurology, Xiangya Hospital, Central South University, Changsha, China.; 4 Faculty of Life and Health Sciences, and Brain Cognition and Brain Disease Institute, Shenzhen Institute of Advanced Technology, Chinese Academy of Sciences, Shenzhen, China.; 5 Innovative Institute of Chinese Medicine and Pharmacy, Shandong University of Traditional Chinese Medicine, Jinan, China.; 6 Department of Laboratory Medicine and Pathology, University of Minnesota Medical School, Minneapolis, Minnesota, USA.; 7 TaiKang Center for Life and Medical Sciences, Wuhan University, Wuhan, China.

**Keywords:** Neuroscience, Parkinson disease

## Abstract

Cerebrotendinous xanthomatosis (CTX), an autosomal recessive disorder characterized by high levels of cholestanol in the blood and accumulation of cholestanol in multiple tissues, especially the brain, often presents in parkinsonism. However, it remains unknown whether cholestanol plays a role in the pathogenesis of sporadic Parkinson’s disease (PD). Here, we show that the levels of serum cholestanol in patients with sporadic PD are higher than those in control participants. Cholestanol activates the protease asparagine endopeptidase (AEP) and induces the fragmentation of α-synuclein (α-syn) and facilitates its aggregation. Furthermore, cholestanol promotes the spreading of α-syn pathology in a mouse model induced by intrastriatal injection of α-syn fibrils. KO of AEP or administration of an AEP inhibitor ameliorates α-syn pathology, degeneration of the nigrostriatal dopaminergic pathway, and PD-like motor symptoms. These results not only indicate that cholestanol contributes to the aggregation and spreading of α-syn by activating AEP but also reveal an opportunity for treating PD with AEP inhibitors.

## Introduction

Parkinson’s disease (PD) is a progressive neurodegenerative disease characterized by both motor and nonmotor features (1). Pathologically, PD is characterized by the loss of dopaminergic neurons in the substantia nigra pars compacta (SNpc) and the formation of Lewy bodies in the remaining neurons. Lewy bodies are predominantly composed of aggregated α-synuclein (α-syn) fibrils (2). During the onset of PD, α-syn forms insoluble fibrils that act as seeds to induce the aggregation of endogenous α-syn in a prion-like manner (3). The etiology and pathogenesis of PD are not fully understood. Cerebrotendinous xanthomatosis (CTX) is an autosomal recessive genetic disease caused by mutations in the *CYP27A1* gene and is characterized by the deposition of cholestanol in the brain, tendons, eye lenses, and other organs (4). The clinical characteristics of CTX include neurological and neuropsychiatric abnormalities, formation of tendon xanthomas, and premature bilateral cataracts (5). Notably, 1 study reported that 23% of patients with CTX have movement disorders, including parkinsonism (6). Other studies reported that 21%–33% of patients with CTX have movement disorders (7, 8). Imaging studies reported decreased uptake of 6-fluoro-l-dopa in the striatum and decreased uptake of dopamine transporter (DAT) in the posterior putamen of patients with CTX, indicating degeneration of the nigrostriatal dopaminergic pathway, which is characteristic of patients with PD (9, 10).

Mutation of the *CYP27A1* gene impairs the synthesis of bile acid, which negatively feeds back to the rate-limiting enzyme CYP7A1, leading to increased production of the intermediate product 7α-hydroxy-4-cholesten-3-one (11). The levels of 7α-hydroxy-4-cholesten-3-one in patients with CTX are 100-fold higher than those in study control participants (12). It shuttles across the blood–brain barrier freely and is metabolized to cholestanol, which is deposited in the brains of patients with CTX (12, 13). This pathway contributes 70% of the cholestanol in the brains of patients with CTX (14). High cholestanol levels are the biomarker for the diagnosis of CTX (15). Interestingly, the content of cholestanol in the substantia nigra of patients with CTX is higher than that of control participants (16). These observations indicate that increased cholestanol levels may trigger the degeneration of the nigrostriatal dopaminergic pathway and progression of parkinsonism. However, the underlying molecular mechanisms remain unknown.

Asparagine endopeptidase (AEP), also known as δ-secretase or legumain (LGMN), is a lysosomal cysteine endopeptidase expressed in plants, blood flukes, ticks, and mammals (17). AEP specifically hydrolyzes the C-terminus of asparagine residues of its substrates, inducing the production of toxic protein fragments (18). Previously, we reported that AEP is activated in an age-dependent manner in the brain and cleaves α-syn at N103, generating an N-terminal α-syn (1-103) fragment that is prone to aggregate and mediates PD-like pathogenesis (19). AEP is also involved in fenpropathrin-induced PD-like symptoms (20). The expression of AEP is promoted by C/EBPβ, a neuroinflammation-related transcription factor (21, 22). We performed a high-throughput screen and identified a selective AEP inhibitor termed compound 11 (CP11). CP11 specifically blocks AEP but not other related cysteine proteases (23).

In the present work, we tested whether cholestanol plays a role in the pathogenesis of sporadic PD. We show that cholestanol activates the C/EBPβ/AEP pathway, induces the fragmentation and aggregation of α-syn, and promotes PD pathogenesis. AEP KO abolishes the detrimental effect of cholestanol on α-syn spreading. In addition, an AEP inhibitor blunts cholestanol-induced α-syn pathology in primary neurons and mouse brains injected with α-syn preformed fibrils (PFFs). These results suggest that cholestanol promotes PD pathology by activating the C/EBPβ/AEP pathway. AEP inhibitors may be a potential treatment for PD, especially for those patients with increased cholestanol levels.

## Results

### Serum cholestanol is increased in patients with sporadic PD.

We first investigated the clinical, pathological, and radiological characteristics of a 47-year-old female patient with CTX who developed parkinsonism. Genetic testing identified compound heterozygous mutations of the *CYP27A1* gene (Supplemental Figure 1A; supplemental material available online with this article; https://doi.org/10.1172/jci.insight.165841DS1). PET scan of the Achilles tendon using ^18^F-2-deoxy-2-fluoro-glucose showed enhanced local metabolic activity (Supplemental Figure 1B). H&E staining of the Achilles tendon sections showed degeneration of the fibrous collagenous tissue, accumulation of cholesterol crystals, hyaline degeneration of muscle tissue, and the appearance of Touton giant cells and foam cells (Supplemental Figure 1C). MRI showed abnormal signals in the bilateral cerebellar hemispheres, midbrain, and bilateral posterior limbs of the internal capsule (Supplemental Figure 2, A and B). Magnetic resonance spectroscopy imaging of the brain showed abnormal signals in the bilateral posterior limbs of the internal capsule (Supplemental Figure 2C). As expected, the levels of cholestanol in the serum of this patient and another patient with CTX were much higher than those of the control participants (Supplemental Figure 2D). PET imaging of the proband showed lower striatal DAT signals than that of the control participants. Neuromelanin-sensitive MRI showed decreased neuromelanin content, suggesting degeneration of the dopaminergic nigrostriatal pathway, which is characteristic of patients with PD (Supplemental Figure 2, E and F). CTX is caused by mutation of the *CYP27A1* gene, which impairs cholesterol metabolism and causes elevated levels of cholestanol. We further investigated the levels of cholestanol in serum samples from 20 sporadic patients with PD with Hoehn and Yahr staging I to II and serum samples from 19 healthy volunteers. Interestingly, the serum levels of cholestanol in patients with PD were higher than those in control participants (Supplemental Figure 2G). Notably, metabolism-related indicators and lipid profiles were not different between the 2 groups (Supplemental Table 1).

### Cholestanol activates the C/EBPβ/AEP pathway and induces cell injury.

**Figure 1:**
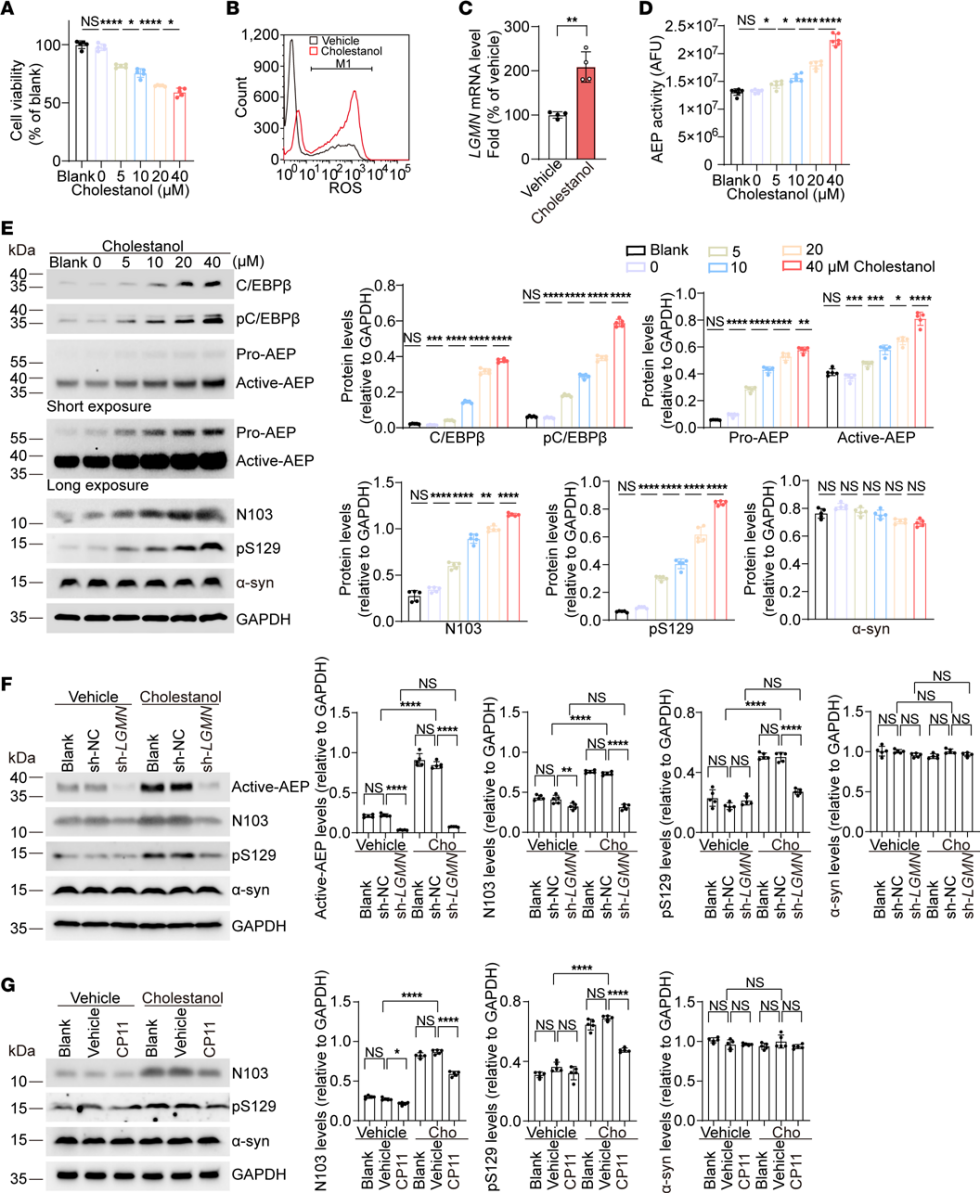
Cholestanol induces cell injury by activating the C/EBPβ/AEP pathway. (**A**) Viability of SH-SY5Y cells exposed to vehicle (0 μM) or cholestanol (5, 10, 20, or 40 μM) for 24 hours (*n* = 5 independent experiments). (**B**) The levels of ROS after exposure to cholestanol (5 μM) for 24 hours. (**C**) mRNA levels of *LGMN* (*n* = 4 independent experiments). (**D**) AEP activity assay (*n* = 6 independent experiments). (**E**) Western blot analysis of C/EBPβ, p-C/EBPβ, AEP, AEP-generated α-syn N103 fragment, p-α-syn, and total α-syn in cholestanol-treated SH-SY5Y cells (*n* = 5). (**F**) Western blots showing the effect of cholestanol (20 μM) on AEP, AEP-generated α-syn N103 fragment, p-α-syn, and total α-syn in SH-SY5Y cells transfected with sh-*LGMN* (*n* = 5). (**G**) Western blots showing the effect of cholestanol (20 μM) on the AEP-generated α-syn N103 fragment, p-α-syn, and total α-syn in SH-SY5Y cells pretreated with CP11 (2 μM) (*n* = 5). Data are presented as mean ± SD. *P < 0.05, **P < 0.01, ***P < 0.005, ****P < 0.001. Student’s *t* test was used in **C**; 1-way ANOVA with Tukey’s multiple comparison was used in **A** and **D**–**G**. AFU, arbitrary fluorescence unit; NC, negative control.

To investigate the effect of cholestanol on cell survival, SH-SY5Y cells were exposed to cholestanol. The cell viability decreased in a concentration-dependent manner (Figure 1A). Furthermore, exposure to cholestanol also decreased the activities of the mitochondrial respiratory chain complexes (mitochondrial complexes II, III, IV, and V) and increased the generation of ROS (Supplemental Figure 3, A–E, and Figure 1B), indicating that cholestanol induces mitochondrial dysfunction and oxidative stress, which are believed to contribute to neural injury in PD. We found that the protease AEP cleaves α-syn at N103, generating the α-syn 1–103 fragment that exerts neurotoxicity (19). The expression of AEP is regulated by the transcription of C/EBPβ (22). Thus, we further tested whether cholestanol regulates the C/EBPβ/AEP signaling pathway.

RT-PCR analysis showed that the levels of *LGMN* were elevated after the SH-SY5Y cells were exposed to 10 μM cholestanol for 24 hours (Figure 1C). In addition, the enzymatic activity of AEP was escalated after exposure to cholestanol (Figure 1D). Western blot analysis confirmed that the levels of total C/EBPβ, phosphorylated C/EBPβ (p-C/EBPβ), AEP, AEP-generated α-syn N103 fragment, and hyperphosphorylated α-syn (p-α-syn) were all increased in cells treated with cholestanol in a concentration-dependent manner (Figure 1E). Notably, the cholestanol-induced increases in α-syn cleavage and hyperphosphorylation were decreased in SH-SY5Y cells after infection with lentiviral shRNA-*LGMN* (sh-*LGMN*)-mediated knocked down *LGMN* (Figure 1F). Similarly, inhibition of AEP by the AEP inhibitor CP11 also attenuated cholestanol-induced increases in α-syn cleavage and hyperphosphorylation (Figure 1G). These results suggest that cholestanol activates the C/EBPβ/AEP pathway.

### Cholestanol promotes α-syn aggregation by activating AEP.

**Figure 2:**
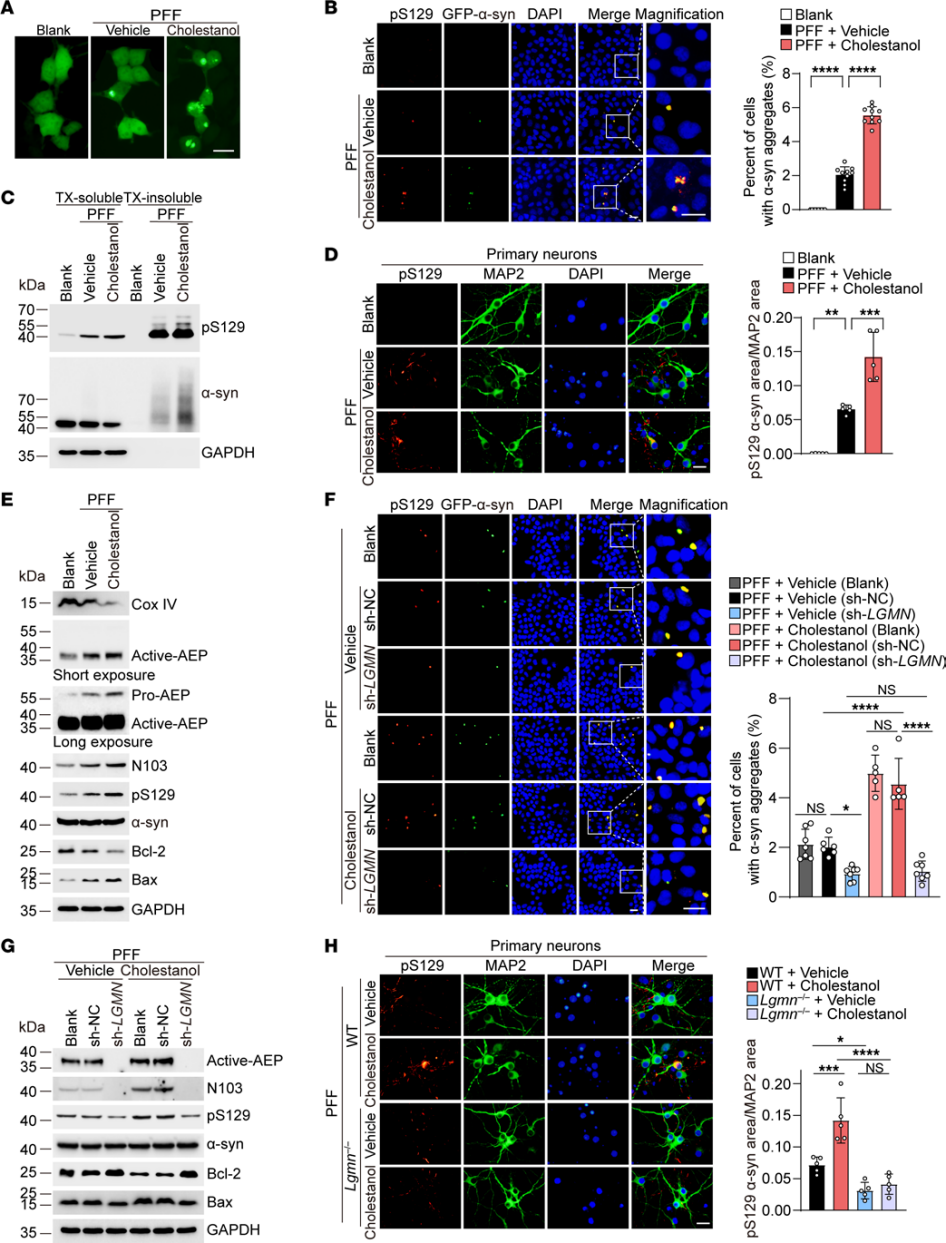
Cholestanol promotes α-syn aggregation by activating AEP. (**A**) Cholestanol promotes α-syn aggregation induced by α-syn PFFs in α-syn–HEK293 cells. Scale bar: 20 μm. (**B**) α-Syn aggregates colocalize with pS129 in α-syn–HEK293 cells. The histogram shows the percentage of cells containing α-syn aggregates (*n* = 10 independent experiments). Scale bars: 20 μm. (**C**) Representative IBs of pS129 and total α-syn in the Triton X-100–soluble and Triton X-100–insoluble fractions in α-syn–HEK293 cells. (**D**) Cholestanol promotes α-syn hyperphosphorylation in primary neurons (*n* = 5 independent experiments). Scale bar: 20 μm. (**E**) Western blot analysis of Cox IV, AEP, AEP-generated α-syn N103 fragment, p-α-syn, total α-syn, Bcl-2, and Bax induced by cholestanol in α-syn–HEK293 cells. (**F**) α-Syn aggregates colocalize with pS129 in α-syn–HEK293 cells transfected with sh-*LGMN*. The histogram shows the percentage of cells containing α-syn aggregates (*n* = 5–7 independent experiments). Scale bars: 20 μm. (**G**) Western blots showing the effect of cholestanol on AEP, AEP-generated α-syn N103 fragment, p-α-syn, total α-syn, Bcl-2, and Bax in α-syn–HEK293 cells transfected with sh-*LGMN*. (**H**) pS129 immunostaining in WT and *Lgmn*^–/–^ primary neurons. The histogram shows the ratio of the pS129 area to the MAP2 area (*n* = 5 independent experiments). Scale bar: 20 μm. Data are presented as mean ± SD. *P < 0.05, **P < 0.01, ***P < 0.005, ****P < 0.001; 1-way ANOVA with Tukey’s multiple comparison test. TX, Triton X-100. NC, negative control.

AEP cleaves α-syn and promotes the propagation of α-syn pathology (19). Thus, we tested the effect of cholestanol on the spreading of α-syn pathology. We used the HEK293 cell line stably transfected with N-terminal GFP-tagged human α-syn (α-syn–HEK293 cells) as reporter cells. The cells were transduced with α-syn PFFs in the presence or absence of cholestanol. Notably, the percentage of cells with aggregates was much higher in cells treated with cholestanol. The aggregates colocalized with pS129, a hallmark of Lewy bodies (Figure 2, A and B). Western blot analysis showed that more pS129-positive α-syn species were found in the Triton X-100–insoluble fractions (Figure 2C and Supplemental Figure 4A).

The effect of cholestanol on the phosphorylation of α-syn was confirmed in primary neurons (Figure 2D). Western blot analysis confirmed that the expression of AEP, α-syn N103 fragment, and p-α-syn were all increased after exposure to cholestanol both in α-syn–HEK293 cells and in primary neurons (Figure 2E and Supplemental Figure 4, B–D). Exposure to α-syn PFFs also induced the activation of AEP and production of α-syn N103. Furthermore, exposure to cholestanol induced mitochondrial degeneration, as illustrated by decreased expression of the mitochondrial marker Cox IV (Figure 2E and Supplemental Figure 4, B, D, and E).

The level of Bcl-2 was decreased, whereas that of Bax was increased in the presence of cholestanol, indicating that cholestanol induces cell toxicity (Figure 2E and Supplemental Figure 4, B and D). The enzymatic activity of AEP was escalated after cholestanol treatment (Supplemental Figure 4F). Knocking down or deleting AEP (sh-*LGMN* or *Lgmn*^–/–^) abolished the effect of cholestanol on α-syn pathology in α-syn–HEK293 cells and primary neurons (Figure 2, F–H, and Supplemental Figure 4G). Moreover, the N103A mutation of α-syn, which blocks AEP-mediated cleavage, also attenuated α-syn aggregation induced by α-syn PFFs and cholestanol (Supplemental Figure 5, A and B). These results indicate that AEP-mediated fragmentation is required for the detrimental effect of cholestanol.

### AEP inhibitor CP11 blunts cholestanol-induced α-syn aggregation in vitro.

**Figure 3:**
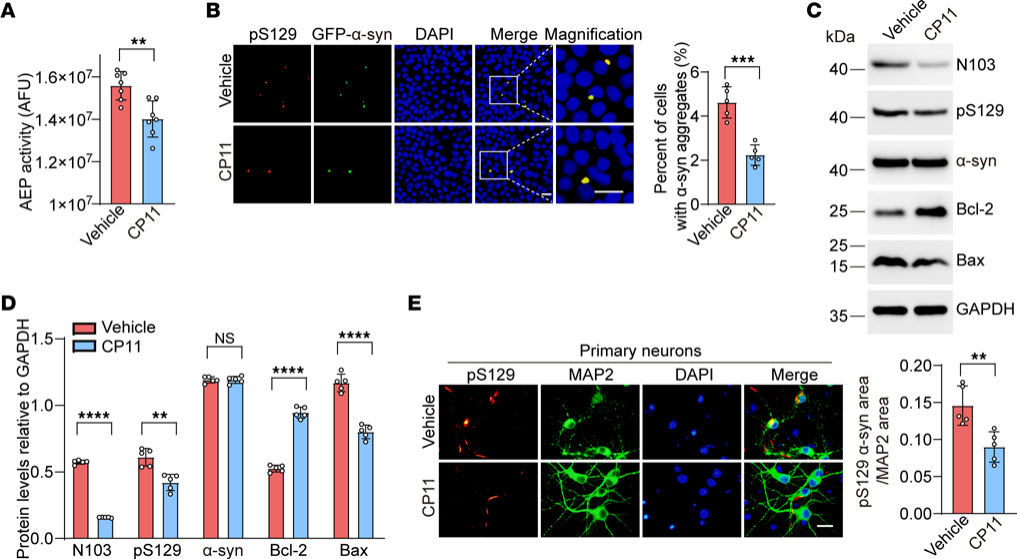
Inhibition of AEP blunts cholestanol-induced α-syn aggregation in vitro. α-Syn–HEK293 cells were pretreated with CP11 (2 μM), followed by transduction with α-syn PFFs (1 μg/mL) and cholestanol (5 μM). (**A**) AEP activity assay (*n* = 7 independent experiments). (**B**) Colocalization of pS129 and α-syn aggregates. The histogram shows the percentage of cells with aggregated α-syn (*n* = 5 independent experiments). Scale bars: 20 μm. (**C**) Western blot analysis of AEP-generated α-syn N103 fragment, p-α-syn, total α-syn, Bcl-2, and Bax. (**D**) Quantitative analysis of protein expression levels (*n* = 5). (**E**) CP11 attenuates the phosphorylation of α-syn in primary neurons. The histogram shows the ratio of the pS129 area to the MAP2 area (*n* = 5 independent experiments). Scale bar: 20 μm. Data are presented as mean ± SD. **P < 0.01, ***P < 0.005, ****P < 0.001; Student’s *t* test. AFU, arbitrary fluorescence unit.

To further confirm the role of AEP in cholestanol-induced α-syn aggregation, α-syn–HEK293 cells were pretreated with the AEP inhibitor CP11 (23), followed by treatment with α-syn PFFs and cholestanol. As expected, cells pretreated with CP11 had decreased AEP enzymatic activity compared with vehicle-treated cells (Figure 3A). The density of pS129-positive α-syn aggregates decreased in cells pretreated with CP11 (Figure 3B). Western blot analysis confirmed that CP11 blocked α-syn cleavage and hyperphosphorylation. CP11 also increased the levels of Bcl-2 and decreased the levels of Bax, supporting a protective effect (Figure 3, C and D). Moreover, CP11 pretreatment attenuated the phosphorylation of α-syn induced by cholestanol in primary neurons (Figure 3E). These results suggest that inhibition of AEP attenuates α-syn aggregation induced by cholestanol.

### Cholestanol promotes α-syn spreading and dopaminergic neuronal loss in vivo.

**Figure 4:**
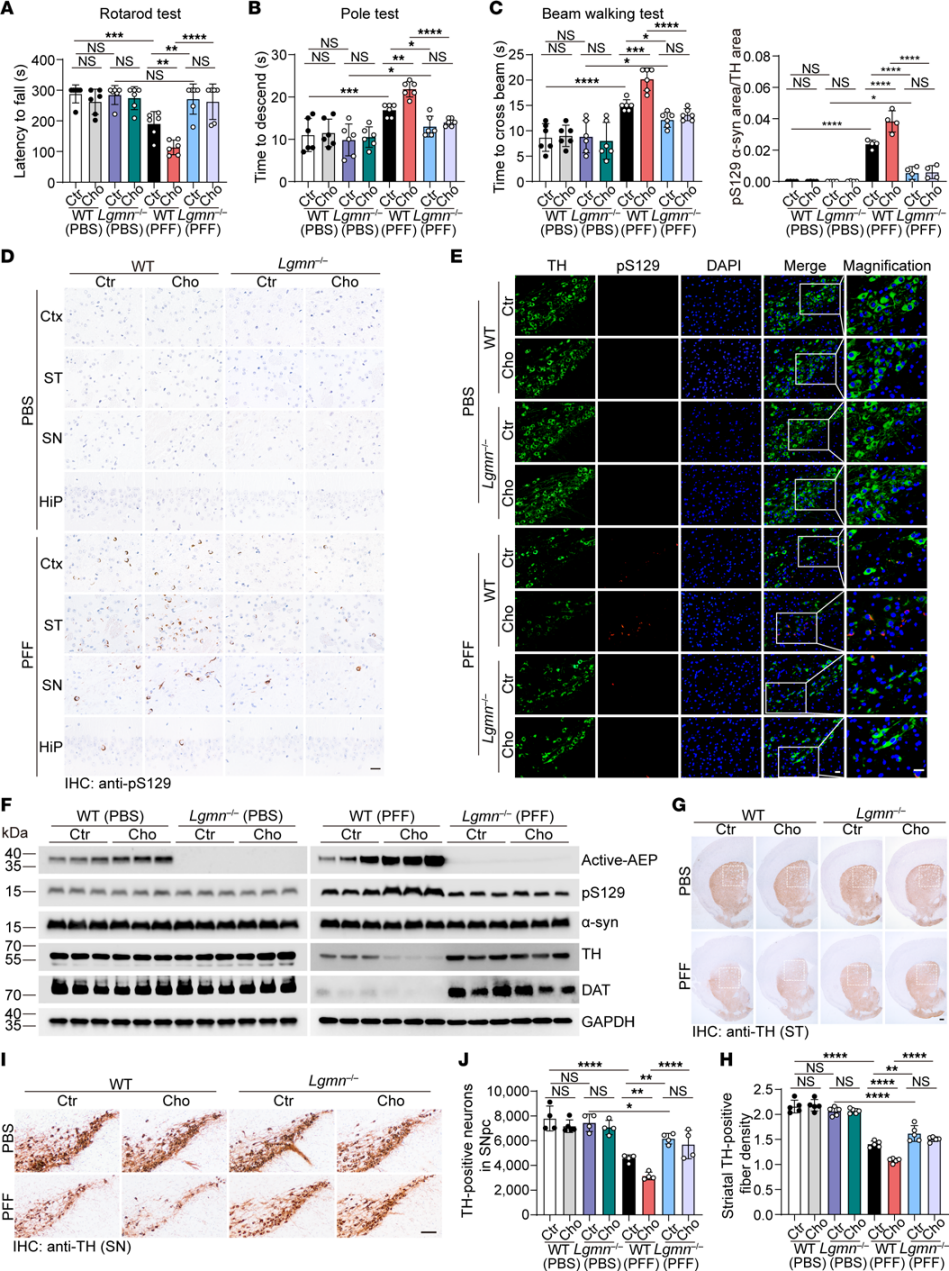
Deletion of AEP attenuates cholestanol-induced α-syn pathology and motor deficits. WT and *Lgmn*^–/–^ mice were fed a cholestanol or chow diet 1 month before stereotaxic injection with α-syn PFFs or PBS. The cholestanol treatment lasted during the whole experiment. (**A**) Rotarod test (*n* = 6). (**B**) Pole test (*n* = 6). (**C**) Beam-walking test (*n* = 6). (**D**) Representative pS129 immunostaining in the cortex, striatum, substantia nigra, and hippocampus of mice sacrificed 6 months after intrastriatal α-syn PFF or PBS injection. Scale bar: 20 μm. (**E**) Double immunostaining of pS129 and TH in the substantia nigra. The histogram shows the ratio of the pS129 area to the TH area (*n* = 4 mice/group). Scale bar: 20 μm. (**F**) Western blot analysis of active-AEP, p-α-syn, total α-syn, TH, and DAT (*n* = 3 mice per group). (**G** and **H**) Representative images of TH-positive fibers in the striatum. The histogram shows the density of TH-positive fibers in the striatum (*n* = 5 mice per group). Scale bar: 200 μm. (**I** and **J**) Representative images of TH-positive neurons in the SNpc. The histogram shows the number of TH-positive neurons in the SNpc (*n* = 4 to 5 mice/group). Scale bar: 100 μm. Data are presented as mean ± SD. *P < 0.05, **P < 0.01, ***P < 0.005, ****P < 0.001; compared by 1-way ANOVA with Fisher’s LSD multiple comparison test. Ctr; control chow diet; Cho, cholestanol; Ctx, cortex; ST, striatum; SN, substantia nigra; Hip, hippocampus.

Intrastriatal injection of α-syn PFFs induces the propagation of α-syn pathology in the mouse brain (24). To test the effect of cholestanol on the propagation of α-syn pathology in vivo, WT mice were fed cholestanol or a chow diet for 1 month and then intrastriatally injected with α-syn PFFs or PBS. Six months after injection, behavioral tests, including the rotarod test, pole test, and beam-walking test, showed that injection of α-syn PFFs induced PD-like movement disorders. Chronic treatment with cholestanol aggravated these behavioral disorders induced by α-syn PFFs. No obvious behavioral abnormalities were observed in mice injected with PBS (Figure 4, A–C). As expected, the contents of cholestanol in the striatum and substantia nigra were much higher in mice fed cholestanol diets than in those that received chow diets (Supplemental Figure 6A).

Immunoreactivity of p-α-syn spread to various brain regions, including the cortex, substantia nigra, and hippocampus, 6 months after injection in mice injected with α-syn PFFs but not in those injected with PBS. Consistent with the in vitro experiments, α-syn pathology was much more severe in the cholestanol-treated mice than in the control mice (Figure 4, D and E, and Supplemental Figure 7A).

Incubation of α-syn PFFs with brain tissue lysates from cholestanol-treated mice induced the formation of the α-syn N103 fragment, indicating that cholestanol promotes the fragmentation of α-syn (Supplemental Figure 7, B and C). Moreover, the levels of C/EBPβ, p-C/EBPβ, AEP, and p-α-syn were strongly upregulated in mice treated with cholestanol, indicating the activation of the C/EBPβ/AEP pathway. The levels of tyrosine hydroxylase (TH) and DAT in the striatum were substantially reduced in mice fed cholestanol compared with mice fed chow diets (Figure 4F, Supplemental Figure 6, B–D, and Supplemental Figure 7, D–H). Furthermore, IHC staining was performed to assess the loss of TH-positive neurons in the SNpc and the TH-positive fibers in the striatum. Injection of α-syn PFFs induced loss of dopaminergic neurons in the substantia nigra and dopaminergic terminals in the striatum, which was exacerbated by cholestanol (Figure 4, G–J). Nissl staining further confirmed cholestanol-exacerbated neurodegeneration in the SNpc (Supplemental Figure 7, I and J). These results suggest that cholestanol activates the C/EBPβ/AEP pathway, which mediates α-syn fragmentation, phosphorylation, and aggregation and promotes the degeneration of the nigrostriatal pathway.

### AEP KO abolishes the promoting effect of cholestanol on α-syn pathology.

To determine whether AEP is required for the effect of cholestanol on the spreading of α-syn pathology, we fed AEP KO (*Lgmn*^–/–^) mice cholestanol or a chow diet and stereotactically injected α-syn PFFs or PBS into the dorsal striatum. Six months after injection, behavioral tests showed that cholestanol enhanced PD-like motor impairments induced by α-syn PFFs in WT mice. We found that the detrimental effects of cholestanol were abolished in *Lgmn*^–/–^ mice. No obvious behavioral abnormalities were observed in mice injected with PBS (Figure 4, A–C).

IHC showed that deletion of AEP abolished the promoting effect of cholestanol on α-syn spreading. The extent of α-syn pathology in the striatum and substantia nigra was attenuated by the deletion of AEP. α-Syn pathologies were also dramatically reduced in TH-positive dopaminergic neurons in *Lgmn*^–/–^ mice (Figure 4, D and E, and Supplemental Figure 7A). Deletion of AEP attenuated α-syn fragmentation, α-syn phosphorylation, and the decrease in TH and DAT in the striatum (Figure 4F and Supplemental Figure 7, B–H). In α-syn PFF-injected WT mice, cholestanol significantly reduced the number of TH-positive cells in the SN and the density of TH-positive fibers in the striatum, whereas deletion of AEP abolished the effect of cholestanol in driving the degeneration of the nigrostriatal dopaminergic pathway (Figure 4, G–J). Nissl staining further confirmed that cholestanol-exacerbated neurodegeneration in the SNpc could be alleviated by the deletion of AEP (Supplemental Figure 7, I and J). These results suggest that deletion of AEP abolishes the effect of cholestanol on α-syn fragmentation, phosphorylation, aggregation, and neurotoxicity.

### CP11 attenuates α-syn pathology in vivo.

**Figure 5:**
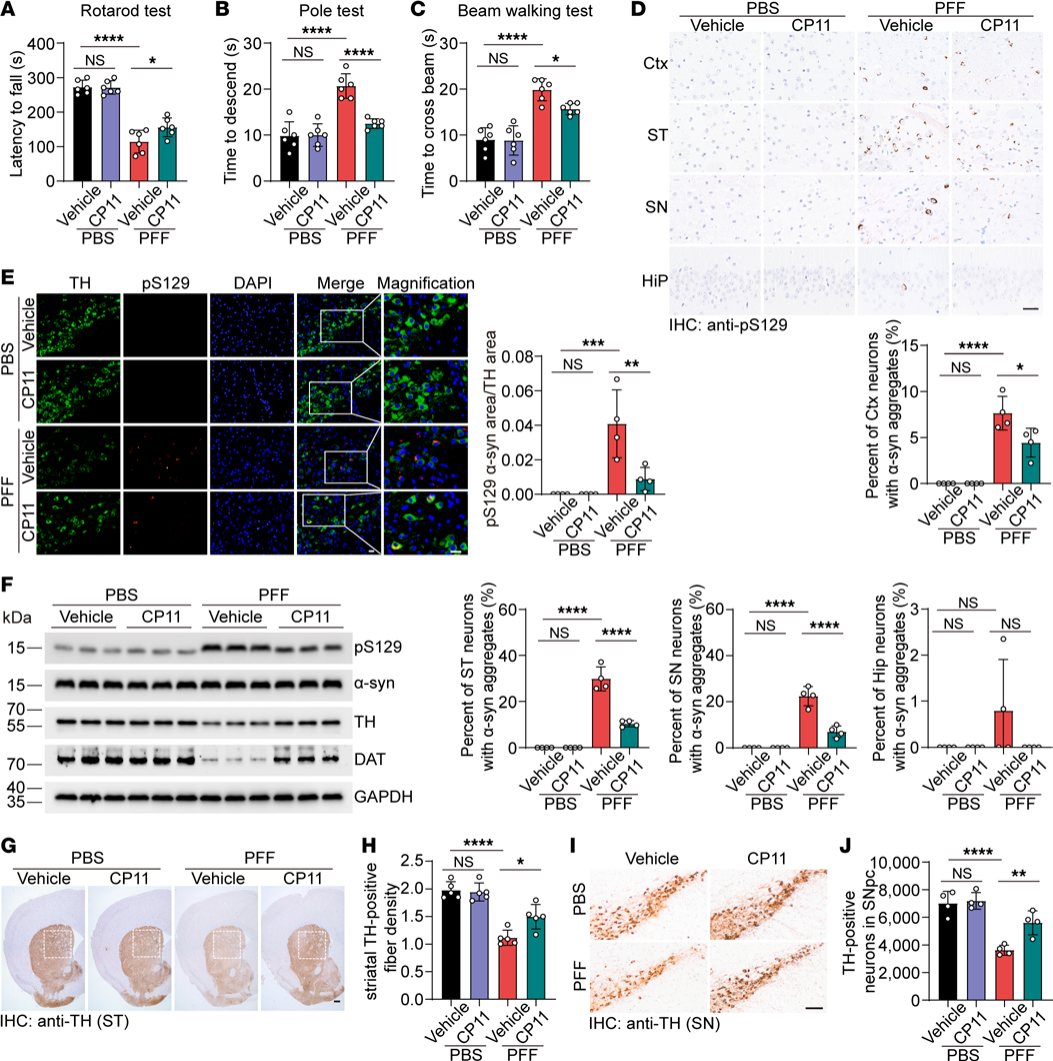
CP11 ameliorates cholestanol-induced α-syn pathology and motor deficits. The mice were injected with CP11 i.p. or vehicle control, fed a cholestanol diet, and then were stereotaxically injected with α-syn PFFs or PBS. (**A**) Rotarod test (*n* = 6). (**B**) Pole test (*n* = 6). (**C**) Beam-walking test (*n* = 6). (**D**) Representative pS129 immunostaining in the cortex, striatum, substantia nigra, and hippocampus of mice sacrificed 6 months after intrastriatal α-syn PFF or PBS injection. The histogram shows the percentage of neurons containing α-syn aggregates (*n* = 4 mice/group). Scale bar: 20 μm. (**E**) Double immunostaining of pS129 and TH in the substantia nigra. The histogram shows the ratio of the pS129 area to the TH area (*n* = 4 mice/group). Scale bar: 20 μm. (**F**) Western blot analysis of p-α-syn, total α-syn, TH, and DAT (*n* = 3 mice/group). (**G** and **H**) Representative images of TH-positive fibers in the striatum. The histogram shows the density of TH-positive fibers in the striatum (*n* = 5 mice per group). Scale bar: 200 μm. (**I** and **J**) Representative images of TH-positive neurons in the SNpc. The histogram shows the number of TH-positive neurons in the SNpc (*n* = 4 mice/group). Scale bar: 100 μm. Data are presented as mean ± SD. *P < 0.05, **P < 0.01, ***P < 0.005, ****P < 0.001; compared by 1-way ANOVA with Tukey’s multiple comparison test. Ctx, cortex; ST, striatum; SN, substantia nigra; Hip, hippocampus.

Because AEP is required for the detrimental effect of cholestanol on the propagation of α-syn pathology, we further investigated the effect of the AEP inhibitor CP11 on the spreading of α-syn pathology in mice injected with α-syn PFFs or PBS. WT mice were injected i.p. with CP11 or vehicle control and concurrently received cholestanol chow starting 1 month before the stereotaxic injection of α-syn PFFs or PBS. Six months after the injection of α-syn PFFs or PBS, mice were subjected to behavioral tests. Results of the rotarod test, pole test, and beam-walking test showed that CP11 alleviated movement disorders induced by α-syn PFFs. No obvious behavioral abnormalities were observed in mice injected with PBS (Figure 5, A–C).

The extent of α-syn pathology in the striatum, substantia nigra, and cortex was also attenuated by CP11. Fewer pS129-positive aggregates were found in the CP11 pretreatment group than in the vehicle control group (Figure 5, D and E). Western blot analysis showed that CP11 attenuated α-syn cleavage and hyperphosphorylation and augmented TH and DAT levels compared with the vehicle control (Figure 5F and Supplemental Figure 8, A–F). Consistently, augmentation of dopaminergic neurons in the substantia nigra and dopaminergic neurites in the striatum was pronounced in mice treated with CP11 (Figure 5, G–J). Nissl staining further confirmed that cholestanol-exacerbated neurodegeneration in the SNpc could be alleviated by inhibition of AEP (Supplemental Figure 8, G and H). These results suggest that inhibition of AEP attenuates the effect of cholestanol on α-syn fragmentation, aggregation, and spreading, relieving the spreading of α-syn pathology and degeneration of the nigrostriatal pathway.

## Discussion

In the present study, we demonstrate that cholestanol activates the C/EBPβ/AEP pathway, stimulating α-syn aggregation and spreading. PET scanning of a patient with CTX showed degeneration of the nigrostriatal dopaminergic pathway, which may underline the clinical observation that patients with CTX are prone to develop PD-like symptoms and pathology (6–10). Cholestanol, the hallmark of CTX, was also found to be elevated in the serum of sporadic patients with PD, suggesting that cholestanol may also contribute to the development of sporadic PD. The aggregation and prion-like spreading of α-syn play a central role in the pathogenesis of PD. α-Syn fibrils can act as templates for the conversion of endogenous soluble α-syn into insoluble hyperphosphorylated aggregates, causing degeneration of the dopaminergic pathway (3, 24). We found that cholestanol promotes the aggregation and spreading of α-syn, causing dopaminergic neuronal injury both in vitro and in vivo.

Previously, we reported that the protease AEP is activated in patients with PD in an age-dependent manner. Active AEP cleaves α-syn and promotes its aggregation, inducing dopaminergic neuronal loss and PD-like motor dysfunction. Although the levels of α-syn (1-103) fragment are not as abundant as the full-length counterparts, they may act as a catalyst to trigger α-syn aggregation (19). The expression of AEP is regulated by the transcription factor C/EBPβ (22). Furthermore, DOPAL, the metabolite of dopamine, activates AEP and subsequently increases α-syn cleavage at N103 (25). The levels of both C/EBPβ and AEP are upregulated in PD brains (19, 26). Thus, the C/EBPβ/AEP signaling pathway plays a key role in the aggregation of α-syn and the onset of PD. Furthermore, activation of the C/EBPβ/AEP pathway is involved in the pathogenesis of Alzheimer’s disease and cancer metastasis (22, 27–30). We also reported that inhibition of mitochondrial complexes I, II, or III escalates ROS and activates the C/EBPβ/AEP pathway (31). Together with the present study, our findings demonstrate that cholestanol induces mitochondrial dysfunction, triggers the activation of the C/EBPβ/AEP pathway, and induces α-syn pathology and neurodegeneration. Elevated cholestanol activates the C/EBPβ/AEP pathway and exacerbates neurodegeneration. The detrimental effect of cholestanol was alleviated by deletion of AEP. These results support that AEP may serve as a novel therapeutic target for PD. Neuronal AEP may act as a biomarker for PD. However, most of the AEP activity in the blood may be derived from peripheral organisms. The development of methods to detect AEP activity in the brain may provide useful information on PD diagnosis.

We identified a small molecular AEP inhibitor, CP11, that specifically blocks the activity of AEP (23). CP11 specifically inhibits the activity of AEP and exerts a protective effect in fenpropathrin-induced parkinsonism as well as in mouse models of Alzheimer’s disease (20, 32). We found that CP11 reduced cholestanol-induced α-syn pathology and degeneration of dopaminergic neurons, suggesting that AEP inhibitors are promising for the treatment of parkinsonism induced by cholestanol. Hence, the results presented here indicate that inhibition of AEP can block α-syn pathology and thus should be evaluated for therapeutic efficacy in treating PD, especially for those patients with higher cholestanol levels.

## Methods

### Abs and reagents.

Abs against the following targets were used: anti-C/EBPβ (H-7) (Santa Cruz Biotechnology, catalog sc-7962); anti–phospho-C/EBPβ (Thr235) (Cell Signaling Technology, catalog 3084); anti-LGMN (D6S4H) (Cell Signaling Technology, catalog 93627); anti–α-syn N103 (homemade, reported previously in ref. 19); anti–phospho-α-syn (Ser129) for immunostaining (BioLegend, catalog 825701); anti–phospho-α-syn (Ser129) (D1R1R) for Western blotting (Cell Signaling Technology, catalog 23706); anti–Cox IV (Abcam, catalog ab16056); anti–Bcl-2 (Cell Signaling Technology, catalog 3498); anti-Bax (Proteintech, catalog 50599-2-Ig); anti-MAP2 (Proteintech, catalog 17490-1-AP); anti–tyrosine hydroxylase (Abcam, catalog ab117112); anti-DAT (Abcam, catalog ab184451); anti–α-syn (Syn211) for Western blotting (Figure 1, E–G; Figure 2, C, E, and G; Figure 3C; and Supplemental Figure 5B) (Thermo Fisher Scientific, catalog MA5-12272); anti–α-syn (D37A6) for Western blotting (Figure 4F, Figure 5F, and Supplemental Figure 4D) (Cell Signaling Technology, catalog 4179); goat anti–rabbit IgG (H+L) highly cross-adsorbed secondary Ab; Alexa Fluor 488 (Invitrogen, catalog A-11034); goat anti–mouse IgG (H+L) cross-adsorbed secondary Ab, Alexa Fluor 594 (Invitrogen, catalog A-11005), goat anti–rabbit IgG (H+L) cross-adsorbed secondary Ab, Alexa Fluor 594 (Invitrogen, catalog A-11012); anti–HRP-conjugated GAPDH (Proteintech, catalog HRP-60004); anti–HRP-conjugated anti–rabbit IgG Abs (Bio-Rad Laboratories, catalog 170-6515); and anti–HRP-conjugated anti–mouse IgG Abs (Bio-Rad Laboratories, catalog 170-6516). Also used were an IHC detection system kit (ZSGB-BIO, catalog PV-6001, PV-6002, ZLI-9019); cholestanol (Sigma-Aldrich, catalog D6128); cholestanol standard (5α-cholestan-3β-ol) for LC-MS (Avanti, catalog 700064P-5MG-E-011); Cell Counting Kit-8 (Dojindo Laboratories, catalog CK04); a ROS assay kit (Nanjing Jiancheng Bioengineering Institute, catalog E004-1-1); electron transport chain complex I activity assay kit (Abcam, catalog ab109721); the following electron transport chain activity assay kits from Solarbio: complex II (catalog BC3235), complex III (catalog BC3245), complex IV (catalog BC0945), and complex V (catalog BC1445); RevertAid first strand cDNA synthesis kit (Thermo Fisher, catalog K1622); Bestar SybrGreen qPCR master mix (DBI Bioscience, catalog DBI-2043); DAPI (BioFroxx); CP11 (Santa Cruz Biotechnology, catalog sc-319780); AEP substrate Z-Ala-Ala-Asn-AMC (Bachem); NP-40 lysis buffer (Beyotime, catalog P0013F); Nissl Stain Kit (methyl violet method) (Solarbio, catalog G1432), RIPA lysis buffer (Fdbio Science, catalog FD009), DMSO (Sigma, catalog D5879), and Triton X-100 (Sigma-Aldrich, catalog T8787).

### Cell culture and treatment.

SH-SY5Y cells, HEK293 cells, and α-syn–HEK293 cells were cultured in DMEM supplemented with 10% FBS at 37°C in an atmosphere containing 5% CO_2_. The isolation and culture of primary cortical neurons were carried out as previously reported (33). Primary neurons were subjected to different treatments on days in vitro (DIV) 7 and used for immunofluorescence experiments on DIV 14. Cholestanol was dissolved and stocked in a 1:1 volume mixture of ethanol and DMSO. CP11 was dissolved and stocked in DMSO. SH-SY5Y cells, α-syn–HEK293 cells, or neurons were pretreated with 2 μM CP11 for 10 hours before undergoing other treatments. The final concentration of DMSO or absolute ethanol in the medium in all groups was below 0.1%.

### Lentiviral infection.

sh-*LGMN* and negative control (sh-NC) were obtained from Obio Technology, as follows: sh-*LGMN* (sense: GCCATGCCTACCAGATCATTC), sh-NC (sense: TTCTCCGAACGTGTCACGT). SH-SY5Y cells or α-syn–HEK293 cells were infected with sh-*LGMN* or sh-NC at an MOI of 10, following the manufacturer’s instructions. Subsequently, knockdown efficiency in cells was assessed by Western blotting.

### Preparation of α-syn PFFs.

Recombinant human α-syn PFFs were prepared as previously described (34), with minor modifications. Briefly, α-syn monomers were incubated in 1.5 mL microfuge tubes for 7 days in PBS (2 mg/mL) at 37°C with shaking at 1000 rpm. Thioflavin T fluorescence was used to confirm the formation of amyloid structures. α-Syn PFFs were then stored at –80°C and sonicated (50 Hz) in an ice-water bath for 40 minutes before transduction. Lipofectamine 2000 was used to transduce α-syn PFFs into α-syn–HEK293 cells, as previously described (35). For the treatment of neurons, 1 μg/mL α-syn PFFs were added to the culture medium.

### Flow cytometry measurement of ROS.

For the quantification of ROS, cells were incubated with 5 μM 2,7-Dichlorofuorescin diacetate fluorescent dye for 20 minutes and washed with PBS. The stained cells were harvested in PBS, and the fluorescence intensities were measured by a BD FACS Calibur. The ROS-positive cells were gated using the forward scatter channel (FSC) and side scatter channel (SSC). Debris and dead cells with low FSC and high SSC were excluded.

### RT-PCR.

Total RNA of SH-SY5Y cells was isolated with TRIzol. RNA (1 μg) was used for cDNA synthesis using the RevertAid first strand cDNA synthesis kit. The amplification reaction was calculated by monitoring the fluorescent dye SybrGreen in a real-time PCR instrument (Bio-Rad Laboratories). Relative quantification of *LGMN* expression was normalized to *GAPDH* and calculated using the comparative Ct method. The following primers were used: *LGMN* forward: GGGTGTTTGGTGTGAGGCT; *LGMN* reverse: TGCTTGCCTCCATCTTCAGG; *GAPDH* forward: TTCTTTTGCGTCGCCAGGTG; *GAPDH* reverse: GGAGGGAGAGAACAGTGAGGC.

### Cell activity assay.

SH-SY5Y cells were seeded in 96-well plates and treated with different concentrations of cholestanol. After treatment, the medium was changed to a mixture of 100 μL of DMEM and 10 μL of CCK8 reagent and incubated in the dark at 37°C. When the reagents turned orange, the plate was read on a microplate reader (SpectraMax, Molecular Devices) at a wavelength of 450 nm.

### Mouse treatment.

The WT C57BL/6J mice were purchased from The Jackson Laboratory. The *Lgmn*^–/–^ mice on a mixed 129/Ola and C57BL/6 background were generated as reported (36). All mice were kept in normal 12-hour light/dark cycles with free access to food and water in the Animal Experiment Center of Renmin Hospital of Wuhan University. All reasonable efforts were made to minimize animal suffering and to use the minimum number of animals necessary to perform statistically valid analysis.

### Stereotaxic injection of α-syn PFFs.

Three-month-old mice were stereotaxically injected in the right hemisphere with 10 μg of α-syn PFFs (2 mg/mL). Sham animals received an equal volume of PBS. The coordinates of the injection were at the dorsal neostriatum (relative to bregma: +2.0 mm lateral, –0.2 mm caudal, –2.7 mm ventral from dura). The α-syn PFFs were injected at 250 μL/min using a microsyringe. The needles were slowly withdrawn 5 minutes after the injection was completed. α-Syn PFFs were sonicated (50 Hz) in an ice bath for 40 minutes before use.

### Feeding of cholestanol.

Cholestanol was formulated in standard chow (AIN-93G-D10012G) at 2/1000 (wt/wt). Mice were fed cholestanol or control chow for 1 month before α-syn PFF or PBS injection and continued until the end of the experiments. The dose of cholestanol was approximately 150 mg/kg/d. Animals were sacrificed 6 months after α-syn PFFs or PBS treatment.

### CP11 treatment.

Mice were administered CP11 i.p. at a dose of 10 mg/kg/d or vehicle starting 1 month before the injection of α-syn PFFs or PBS for 2 months. CP11 was first dissolved in DMSO and then diluted in corn oil before use. The control group received an equivalent volume of corn oil containing 0.4% DMSO.

### Behavioral tests.

In the rotarod test, mice were placed on a spinning rod and trained for 3 consecutive days, 3 times a day, 5 minutes each time, at a speed of 5–25 rpm with an acceleration of 1 rpm every 5 seconds. There were 30-minute intervals after each training. The test began 1 hour after the last training. The interval between the rod to start until the mice fell was recorded, and each trial had a cutoff limit of 300 seconds.

In the pole test, mice were placed head up on the top of a vertical, rough wooden pole (45 cm long, 1 cm diameter) and waited for autonomous descent. Mice were trained for 2 consecutive days, 3 times a day. The test began after the last training. The time from turning to crawling to the ground was recorded, and each trial had a cutoff limit of 30 seconds.

The beam-walking test was performed to assess motor coordination and balance. A beam (80 cm long and 1.6 cm wide) was placed 50 cm above the floor. The mice were placed on 1 end of a wooden pole and they crawled to the other end autonomously. Mice were trained 3 times per day for 2 days before the formal test. A wooden pole with a length of 80 cm and a width of 0.9 cm was used in the formal test. The duration of the mice crawling on the wooden pole for 50 cm was recorded, and each trial had a cutoff limit of 30 seconds.

### Western blot.

Cells and mouse striatum tissues were lysed in NP-40 or RIPA lysis buffer supplemented with protease inhibitor and protein phosphorylase inhibitor and then lysed on ice for 30 minutes. After being centrifuged at 13,000*g* for 10 minutes at 4°C, the supernatants were boiled in SDS loading buffer. Proteins were separated by 10% SDS-PAGE, transferred to a nitrocellulose membrane, blocked, and then incubated with primary Ab overnight at 4°C. Membranes were washed, incubated with secondary Abs, washed, and imaged using ECL.

### Immunofluorescence.

To detect the phosphorylation of α-syn and mitochondrial enrichment in α-syn–HEK293 cells, the cells were fixed in 4% paraformaldehyde for 10 minutes and permeated in 1% Triton X-100 for 20 minutes. Cells were washed with PBS, blocked with 3% BSA for 30 minutes, and then incubated with anti-pS129 (1:500) or rabbit anti–Cox IV (1:500) primary Ab overnight at 4°C. To detect the phosphorylation of α-syn in primary neurons, neurons were fixed in 4% paraformaldehyde for 10 minutes, permeated in 0.1% Triton X-100 for 5 minutes, blocked with 3% BSA for 30 minutes, and then incubated with a mixture of anti-pS129 (1:500) and anti-MAP2 (1:500) primary Abs overnight at 4°C. After being washed with PBS, the cells were incubated with secondary Abs conjugated to Alexa Fluor 488 or Alexa Fluor 594 for 2 hours at room temperature in the dark. Then, the cells were washed with PBS, followed by nuclear staining with DAPI. Pictures were taken under a fluorescence microscope (Olympus, catalog IX73).

The cells with GFP- and pS129-positive aggregates around the nucleus were considered to be cells with α-syn aggregates. We calculated the percentage of cells with α-syn aggregates out of the total cells in each visual field. To assess the levels of mitochondrial enrichment, the fluorescence intensity (expressed as arbitrary fluorescence units) of the mitochondrial marker Cox IV in each visual field was quantified by ImageJ software. To assess the α-syn phosphorylation levels in primary neurons, the pS129- and MAP2-positive areas were quantified by ImageJ software, and the ratios between pS129- and MAP2-positive areas were used for statistical analysis.

### IHC and immunofluorescence.

Mice were anesthetized, sacrificed, and intracardially perfused with saline and then with 4% paraformaldehyde. The brains were isolated and immersed in 4% paraformaldehyde overnight. Then, the brains were embedded and cut into 4 μm slices. Sections were dewaxed in xylene, rehydrated through an ethanol gradient into water, and then placed in antigen-retrieval buffer (0.1 M sodium citrate, pH 6.0) for 20 minutes at 94°C.

For IHC staining, sections were additionally incubated with hydrogen peroxide for 10 minutes to reduce nonspecific background staining caused by endogenous peroxidase. Then, the sections were blocked with 3% BSA for 30 minutes, followed by incubation with anti-pS129 (1:500) or anti-TH (1:500) primary Abs overnight at 4°C. The signals were developed using the IHC detection kit. For immunofluorescence staining, the slides were processed as described for the IHC procedure.

To illustrate the propagation of α-syn pathology in different brain regions in mice, the percentages of neurons with pS129-positive α-syn aggregates out of the total neurons in each visual field were used for statistical analysis. To assess α-syn phosphorylation levels in TH-positive neurons, pS129- and TH-positive areas were quantified using ImageJ software, and their ratio was used for statistical analysis.

### Dopaminergic neuron counting.

Dopaminergic neuron counting was performed as previously described (35). Briefly, the mouse substantia nigra area was continuously sliced into 4 μm–thick paraffin sections. The thickness of every 6 sections was 24 μm, which is close to the average diameter of the dopaminergic neuron cell bodies. One section was taken from every 6 sections for TH staining. The sum of the cell numbers of 20~25 sections involved was taken to be the number of dopaminergic neurons in a mouse.

### Optical densitometry analysis.

The intensity of striatal TH-positive fibers was measured by optical densitometry in TH immune-stained mouse brain sections from the same striatum area. Striatal sections ipsilateral to the injection side were captured for quantification. Optical densitometry was measured using ImageJ software (NIH).

### Nissl staining.

The neuron number in the mouse SNpc area was also determined according to the manufacturer’s instructions for the Nissl Stain Kit. Mouse brain sections were dewaxed in xylene, rehydrated through an ethanol gradient, and then incubated with methyl violet solution for 20 minutes. Sections were washed with distilled water and differentiated with Nissl differentiation for approximately 8 seconds until most of the staining solution was eliminated. Then, sections were dehydrated by absolute alcohol and were transparentized with xylene. Slices were mounted on slides with neutral balsam and then observed and imaged under a light microscope (Olympus, catalog IX73). We counted Nissl-stained neurons in the SNpc from 4 sections at the same location from 4 brains for each group.

### Measurement of AEP activity and mitochondrial respiratory chain complex activities.

AEP activity was measured according to a previous study (19), with minor modifications. Cell lysates (10 μL) were incubated in 90 μL of AEP buffer (50 mM sodium acetate anhydrous, 0.5% Triton X-100 and 5 mM DTT, pH 6.0) containing 10 μM AEP substrate in a 96-well plate. 7-Amino-4-methylcoumarin released by substrate cleavage was monitored at 460 nm in a SpectraMax i3X microplate reader (Molecular Devices) at 37°C for 1 hour in kinetic mode for 5 minutes. Activities of the mitochondrial respiratory chain complexes were determined spectrophotometrically according to the manufacturer’s manuals for the Mitochondrial Respiratory Chain Complex Activity Assay Kits.

### LC-MS to detect cholestanol.

For human serum samples, 20 μL of each sample was transferred to a 1.5 mL tube. After the addition of 80 μL of methanol, the samples were vortexed for 30 seconds, homogenized at 40 Hz for 4 minutes, sonicated for 5 minutes in an ice-water bath, and then centrifuged at 15,294 *g* for 15 minutes at 4°C. For mouse striatum and substantia nigra tissues, each sample (20 mg) was precisely weighed and transferred to a 2 mL tube. After adding 500 μL methanol, the samples were vortexed for 30 seconds, homogenized at 40 Hz for 4 minutes, and sonicated for 5 minutes in an ice-water bath. The homogenate and sonication cycle were repeated 3 times, and then the samples were centrifuged at 15,294 *g* for 15 minutes at 4°C. Supernatant (40 μL) was transferred to an autosampler vial for liquid chromatography with tandem mass spectrometry analysis. The injection volume was 1 μL. Ultra-high performance liquid chromatography (UHPLC) separation was carried out using an Agilent 1290 Infinity II series UHPLC System (Agilent Technologies) equipped with Agilent ZORBAX Eclipse Plus C18 (2.1 mm ×50 mm; 1.8 μm). The contents of cholestanol in mouse tissues were expressed as nanograms per milligram of tissue and as micromolar per liter in human serum samples.

### Statistics.

All data were analyzed using GraphPad software, version 8.3.0. Data were expressed as mean ± SD. The differences between 2 groups were assessed by the 2-tailed Student’s *t* test. The differences between multiple groups were assessed by 1-way ANOVA with Tukey’s multiple comparisons test or Fisher’s LSD multiple comparison test. *P* < 0.05 was considered statistically significant.

### Study approval.

All animal experimental procedures were approved by the Laboratory Animal Welfare Ethical Committee (IACUC) of Renmin Hospital of Wuhan University (approval 20210117). For studies involving humans, participants gave verbal informed consent or signed informed consent.

### Data availability.

Supporting data values for each figure are made available in the Supporting Data Values file.

## Author contributions

Zhentao Zhang conceived and supervised the project. TY performed most of the experiments. SN, ML, and JH collected the clinical data. LB, XN, HF, JG, BT, Zhaohui Zhang, KY, HJ, and LC helped with data analysis and interpretation. All authors read and approved the submission of the manuscript.

## Supplementary Material

Supplemental data

Supporting data values

## Version 1 11/08/2023

Electronic publication
