# Supplementary material for: Cholestanol accelerates α-synuclein aggregation and spreading by activating asparagine endopeptidase
Source: JCI Insight. 2023 Nov 8;8(21):e165841. doi: 10.1172/jci.insight.165841 (PMC10721279; doi:10.1172/jci.insight.165841)
Supplement: Supplemental data [file jciinsight-8-165841-s221.pdf]

Supplemental Figure 1

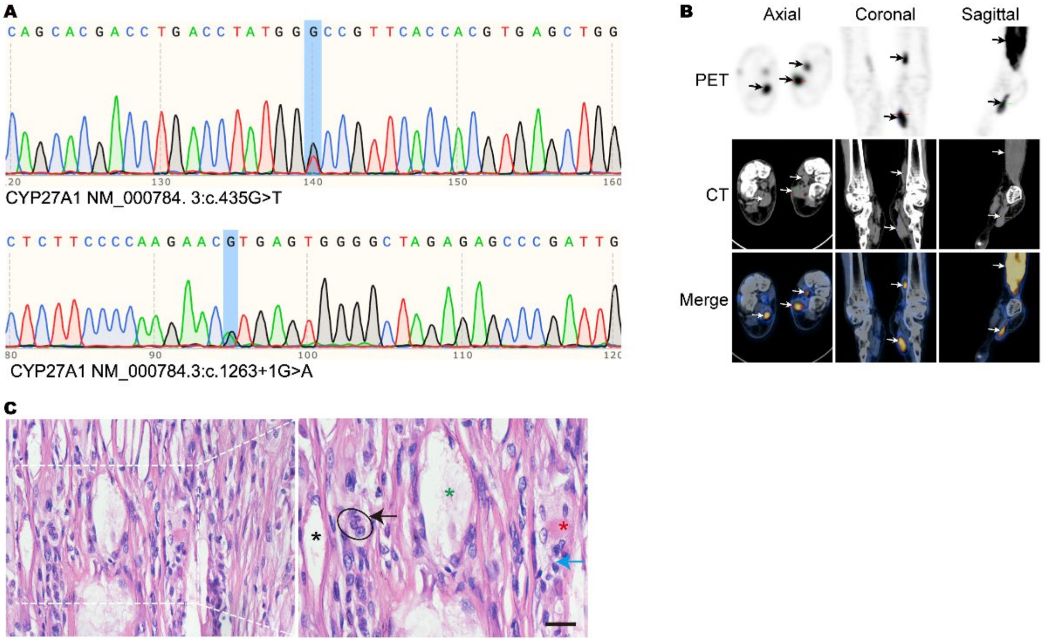

Supplemental Figure 1. Characterization of the CTX patient. (A) Genetic examination of the *CYP27A1* gene. **(B)** Axial, coronal, and sagittal planes of positron emission tomography (PET) and computed tomography (CT) imaging of the Achilles tendon. Arrows indicate high radioactivity in PET and fusion windows and abnormal soft-tissue thickening in CT windows. **(C)** Tendon hematoxylin-eosin staining on microscopic examination. Scale bar, 20 µm. Black star, degeneration of fibrocollagenous tissue. Green star, cholesterol crystals. Red star, hyaline degeneration of muscle tissue. Black arrow, Touton giant cells. Blue arrow, foam cell.

Supplemental Figure 2

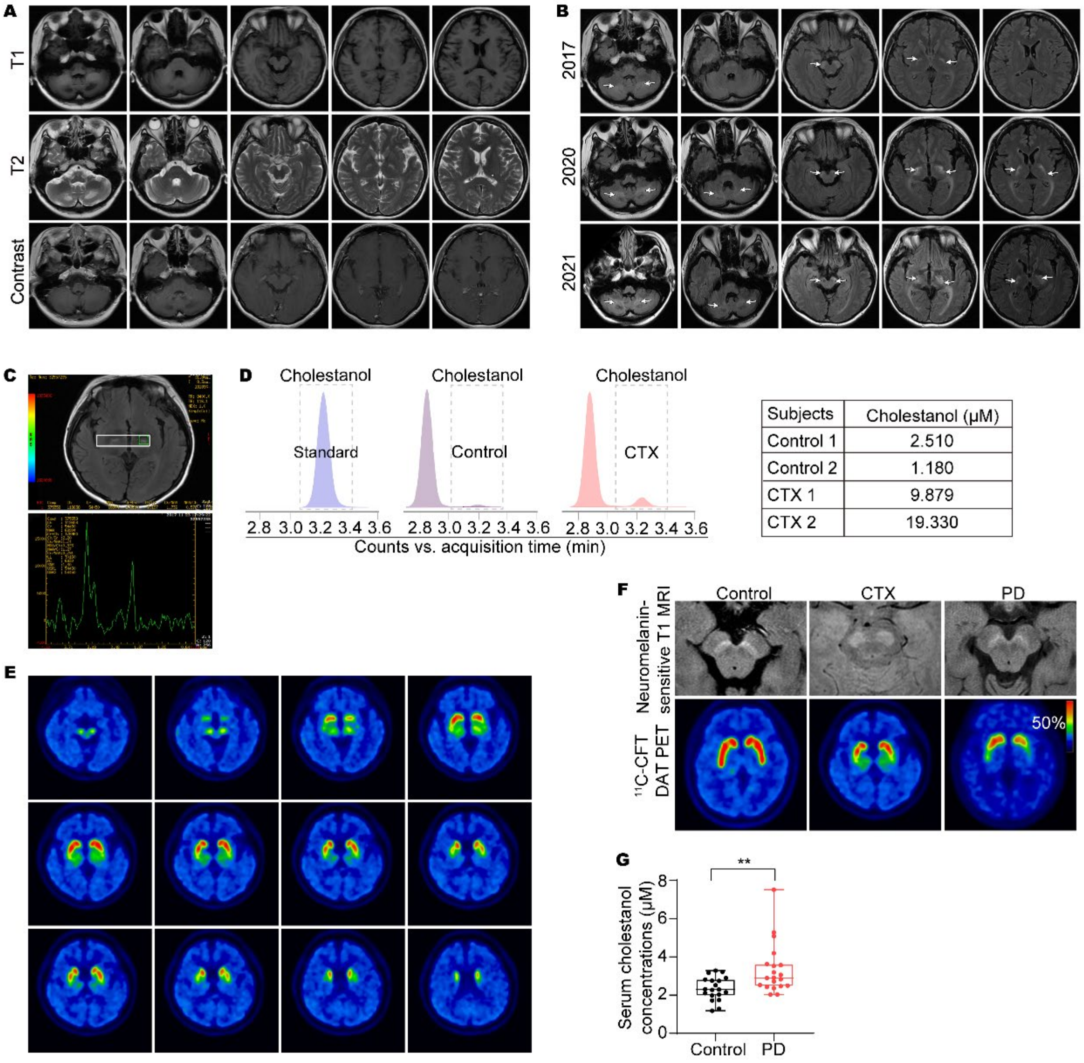

Supplemental Figure 2. Additional characterization of the CTX patient. (A) Magnetic resonance imaging (MRI) of the brain in 2017 showing abnormal signals in the bilateral cerebellar hemispheres, midbrain, and bilateral posterior limbs of the internal capsule. **(B)** T2 fluid-attenuated inversion recovery (FLAIR) MRI of the brain in 2017, 2020, and 2021. Arrows indicate abnormal signals in the bilateral cerebellar hemispheres, midbrain, and bilateral posterior limbs of the internal capsule. **(C)** Magnetic resonance spectroscopy (MRS) imaging of the brain showing abnormal signals in the bilateral posterior limbs of the internal capsule. **(D)** Serum cholestanol

levels were measured by LC-MS. The peaks at approximately 3.2 min represent cholestanol. **(E)** ^{11}C-CFT PET dopamine transporter imaging of the brain. **(F)** Neuromelanin-sensitive magnetic resonance imaging and ^{11}C-CFT PET dopamine transporter imaging in a healthy volunteer, a CTX patient, and a PD patient. **(G)** Serum cholestanol levels in control subjects (n = 19) and PD patients (n = 20). Data are presented as means ± SD; $^{**}P < 0.01$; Student's *t*-test.

Supplemental Figure 3

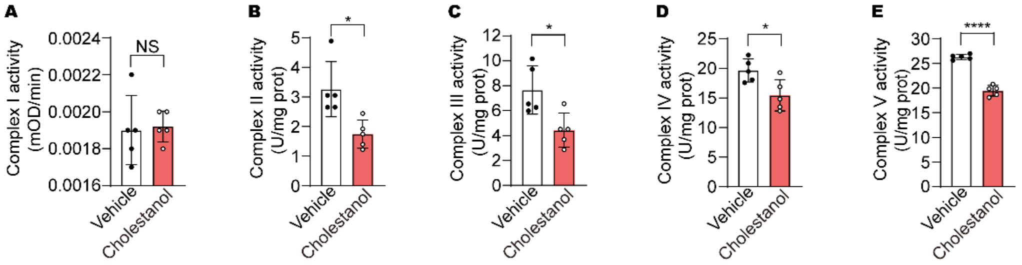

Supplemental Figure 3. Cholestanol induces mitochondrial dysfunction. (A-E) Mitochondrial complex I, II, III, IV, and V activities of SH-SY5Y cells exposed to cholestanol (5 μM) for 24 h (n = 5 independent experiments). Data are presented as means ± SD; ns: not significant; **P* < 0.05, *****P* < 0.001; Student's *t*-test.

Supplemental Figure 4

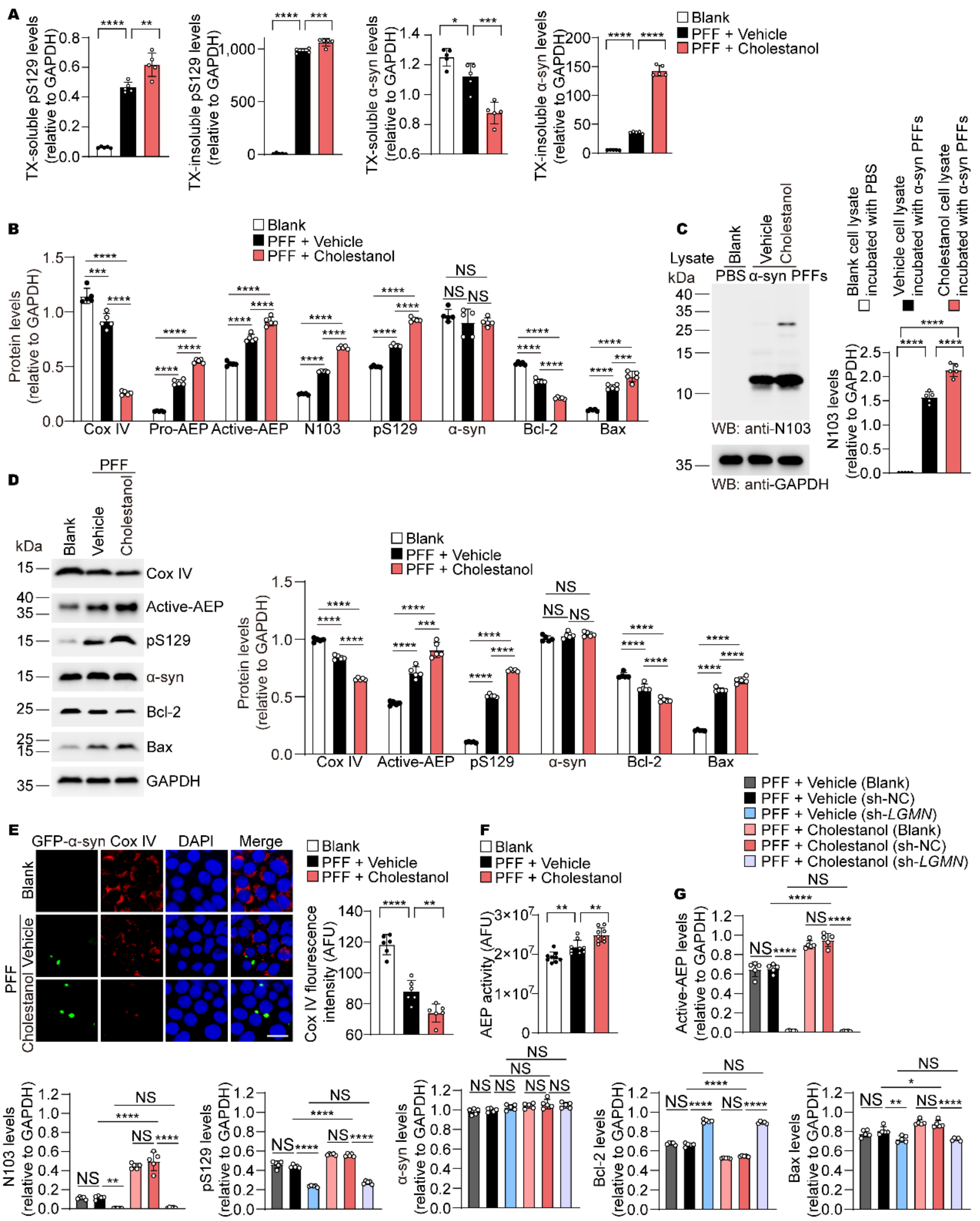

Supplemental Figure 4. Cholestanol activates AEP and promotes the deposition of α-syn. (A) Quantitative analysis of TX-soluble and TX-insoluble p-α-Syn and total α-syn expression levels (n = 5). **(B)** Quantitative analysis of Cox IV, AEP, AEP-generated α-syn N103 fragment, p-α-Syn, total α-syn, Bcl-2, and Bax expression levels (n = 5).

(C) Cholestanol promotes the generation of the α-syn N103 fragment. α-Syn PFFs were incubated with lysates from WT primary neurons treated with cholestanol or vehicle. Western blot analysis of α-syn N103 fragment. Quantitative analysis of the α-syn N103 fragment (n = 5). **(D)** Western blot analysis of Cox IV, AEP, p-α-syn, total α-syn, Bcl-2, and Bax induced by cholestanol in WT primary neurons (n = 5). **(E)** α-syn-HEK293 cells were immunostained with the mitochondrial marker Cox IV. The histogram shows the fluorescence intensities of Cox IV (n = 6 independent experiments). Scale bar, 20 μm. **(F)** AEP activity assay (n = 9 independent experiments). **(G)** Quantitative analysis of AEP, AEP-generated α-syn N103 fragment, p-α-Syn, total α-syn, Bcl-2, and Bax induced by cholestanol in α-syn-HEK293 cells transfected with sh-*LGMN* (n = 5). Data are presented as means ± SD; ns: not significant; *$P < 0.05$, **$P < 0.01$, ***$P < 0.005$, ****$P < 0.001$; compared by one-way analysis of variance (ANOVA) with Tukey's multiple comparison test. TX, Triton X-100; AFU, arbitrary fluorescence units.

Supplemental Figure 5

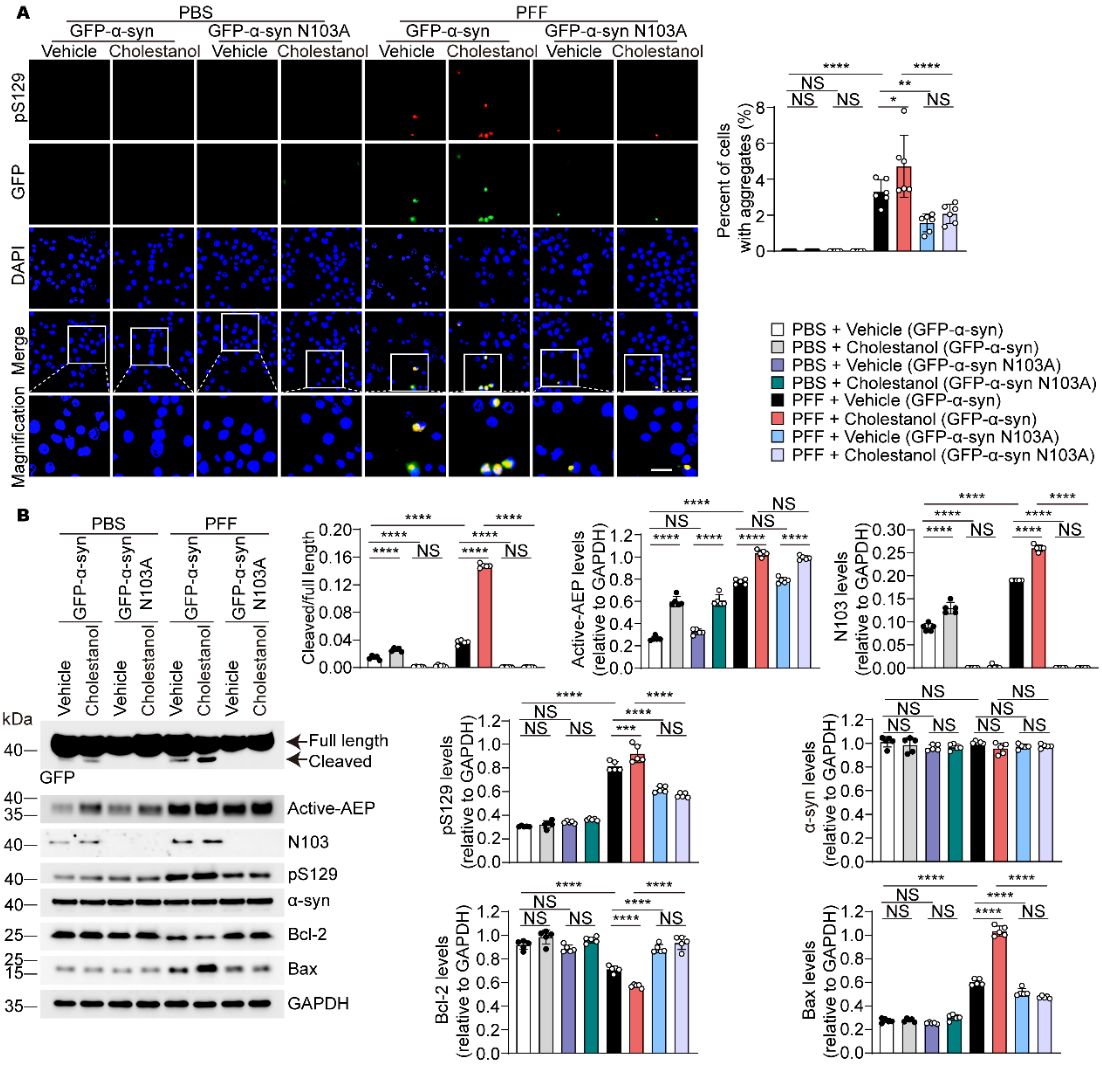

Supplemental Figure 5. Fragmentation of α-syn is important in cholestanol-induced α-syn aggregation. Mutation of N-terminal GFP-tagged human α-syn in N103 attenuated cholestanol-induced α-syn pathology in HEK293 cells transduced with α-syn PFFs. **(A)** α-Syn aggregates colocalize with pS129. The histogram shows the percentage of cells containing aggregates (n = 5 to 6 independent experiments). Scale bars, 20 μm. **(B)** Western blot analysis of GFP, AEP, AEP-generated α-syn N103 fragment, p-α-Syn, total α-syn, Bcl-2, and Bax (n=5). Data are presented as means ± SD; ns: not significant; *$P < 0.05$, **$P < 0.01$, ***$P < 0.005$, ****$P < 0.001$; compared

by one-way analysis of variance (ANOVA) with Tukey's multiple comparison test.

Supplemental Figure 6

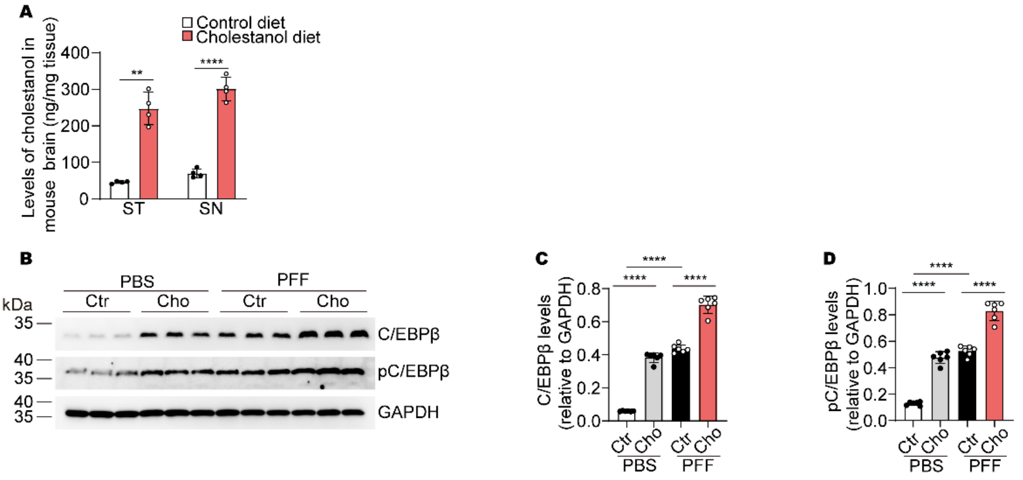

Supplemental Figure 6. Cholestanol activates C/EBPβ in vivo. (A) LC-MS analysis of cholestanol. The histogram shows cholestanol content in the striatum and substantia nigra (n = 4 mice per group). **(B-D)** Western blot analysis of C/EBPβ and p-C/EBPβ in wild-type mice fed control diet or cholestanol (n = 6). Data are presented as means ± SD; $^{**}P < 0.01$, $^{****}P < 0.001$; compared by Student's *t*-test between two groups and one-way analysis of variance (ANOVA) with Tukey's multiple comparison test between multiple groups. ST, striatum; SN, substantia nigra; Ctr; control chow diet; Cho, cholestanol.

Supplemental Figure 7

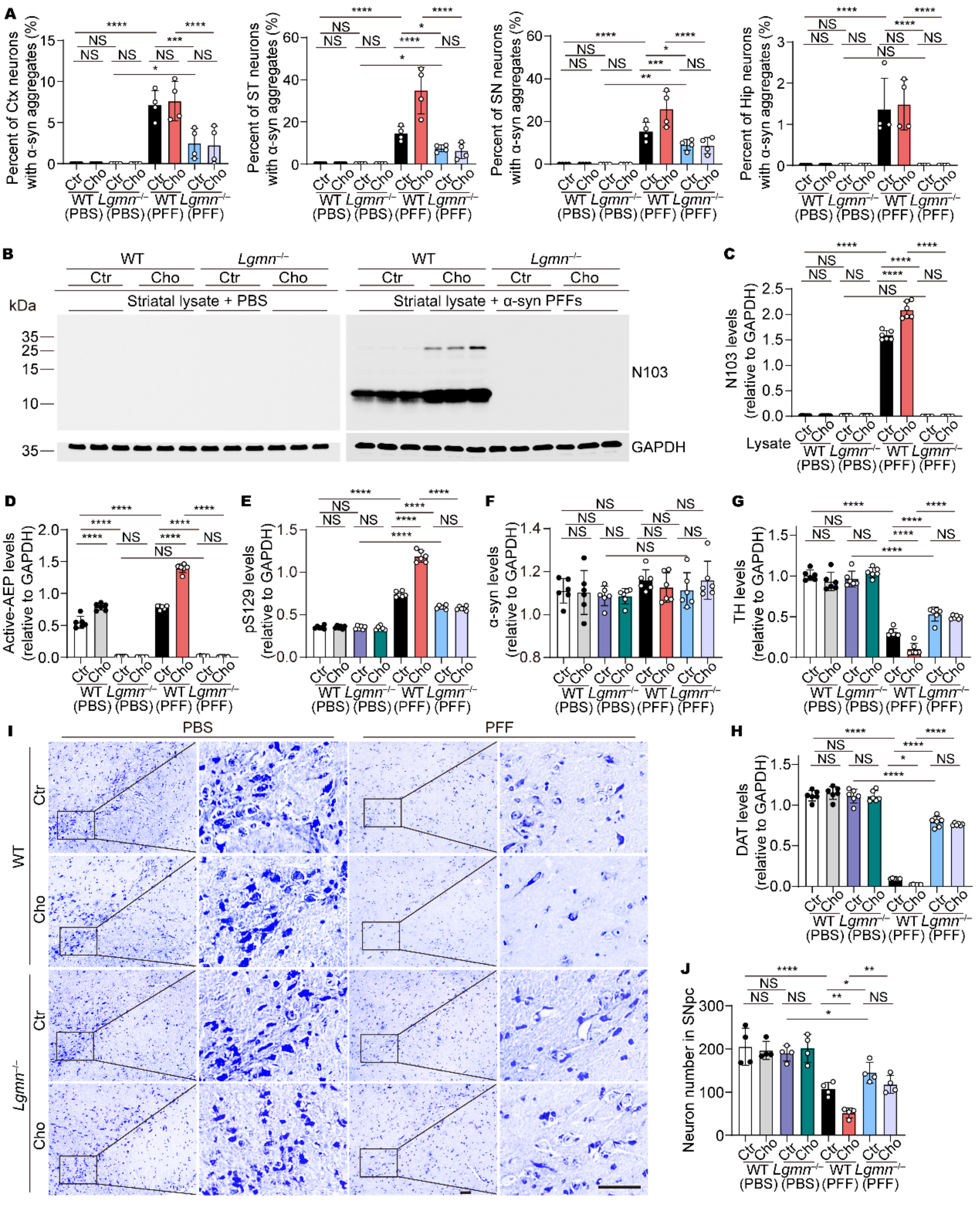

Supplemental Figure 7. Deletion of AEP abolishes the detrimental effect of cholestanol. (A) The histogram shows the percentage of neurons containing α-syn aggregates in the cortex, striatum, substantia nigra, and hippocampus of wild-type (WT) and AEP knockout (*Lgmn*$^{-/-}$) mice sacrificed 6 months after intrastriatal α-syn PFFs or PBS injection (n = 4 mice per group). **(B)** Deletion of AEP attenuated the generation of

the α-syn N103 fragment. PBS or α-syn PFFs were incubated with brain lysates from wild-type (WT) and AEP knockout (*Lgmn*$^{-/-}$) mice fed with cholestanol or a control chow diet. The levels of α-syn N103 fragment were analyzed by Western blot (n = 3 mice per group). **(C)** Quantitative analysis of the α-syn N103 fragment (n = 6). **(D-H)** Quantitative analysis of active-AEP, p-α-Syn, total α-syn, TH, and DAT expression levels (n = 6). **(I)** Nissl staining in the SNpc. **(J)** Quantification of Nissl staining in the SNpc (n = 4 mice per group). Scale bar, 50 μm. Data are presented as means ± SD; ns: not significant; **P* < 0.05, ***P* < 0.01, ****P* < 0.005, *****P* < 0.001; compared by one-way analysis of variance (ANOVA) with Fisher's LSD multiple comparison test. Ctr, control chow diet; Cho, cholestanol; Ctx, cortex; ST, striatum; SN, substantia nigra; Hip, hippocampus.

Supplemental Figure 8

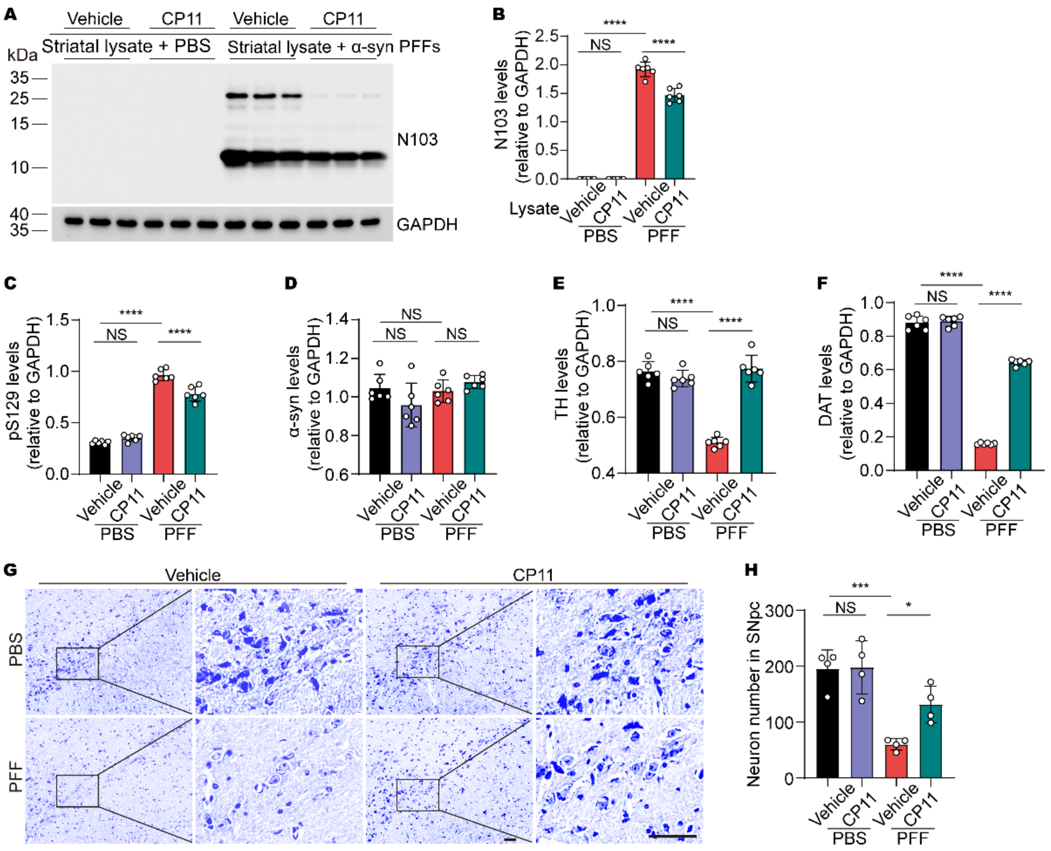

Supplemental Figure 8. AEP inhibitor attenuates the detrimental effect of cholestanol. (A) CP11 reduced the generation of the α-syn N103 fragment. PBS or α-syn PFFs were incubated with brain lysates from mice treated with CP11 or vehicle. The levels of α-syn N103 fragment were analyzed by Western blot (n = 3 mice per group). **(B)** Quantitative analysis of the α-syn N103 fragment (n = 6). **(C-F)** Quantitative analysis of p-α-Syn, α-syn, TH, and DAT expression levels (n = 6). **(G)** Nissl staining in the SNpc. **(H)** Quantification of Nissl staining in the SNpc (n = 4 mice per group). Scale bar, 50 μm. Data are presented as means ± SD; ns: not significant; *P < 0.05, ***P < 0.005, ****P < 0.001; compared by one-way analysis of variance (ANOVA) with Tukey's multiple comparison test. CP11, compound#11.

Table

Supplemental Table 1. Characteristics of PD patients and control subjects.

Supplemental Table 1. Characteristics of PD patients and control subjects

	Control subjects (n = 19)	PD patients (n = 20)	*p* value[a]
**Clinical characteristics**			
Age	67.00 ± 8.93	67.15 ± 5.43	0.950
Gender (m/f)	7/12	9/11	/
H&Y stages	/	1.45 ± 0.43	/
**Metabolic statuses**			
Systolic blood pressure (mmHg)	126.50 ± 13.12	133.50 ± 12.53	0.100
Diastolic blood pressure (mmHg)	74.84 ± 6.73	75.00 ± 12.26	0.960
Fasting blood glucose (mmol/L)	4.93 ± 0.83	5.20 ± 1.42	0.841
Waist circumference	unknown	unknown	/
**Lipid profiles**			
TC (mmol/L)	3.96 ± 0.77	4.19 ± 1.01	0.435
TG (mmol/L)	1.19 ± 0.52	1.05 ± 0.30	0.652
HDL-C (mmol/L)	1.15 ± 0.24	1.26 ± 0.31	0.211
LDL-C (mmol/L)	2.14 ± 0.65	2.25 ± 0.84	0.640

[a]**:** The statistically significant difference of continuous variables between control subjects and PD patients was based on Student's *t*-test, and the data were presented as means ± SD. m/f indicates the ratio of the number of males to the number of females.

Abbreviations: *PD* Parkinson's disease, *H&Y stages* Hoehn and Yahr stages, *TC* total cholesterol, *TG* triglyceride, *HDL-C* high-density lipoprotein cholesterol, *LDL-C* low-density lipoprotein cholesterol
